# Improving micro-grid management: A review of integration of supercapacitor across different operating modes

**DOI:** 10.1016/j.heliyon.2025.e42178

**Published:** 2025-01-23

**Authors:** Mohamed El-Sayed M. Essa, Mohammed Fouad Ali, Elwy E. El-kholy, Mohammed Amer, Mahmoud Elsisi, Uzair Sajjad, Khalid Hamid, Hilmy El-sayed Awad

**Affiliations:** a Electrical Power and Machines Dept., Institute of Aviation Engineering and Technology, Egyptian Aviation Academy, Ministry of Civil Aviation, Imbaba Airport, Giza, Egypt; b Department of Electrical Engineering, Faculty of Technology and Education, Helwan University, Egypt; c Institute of Aviation Engineering and Technology, Egyptian Aviation Academy, Ministry of Civil Aviation, Imbaba Airport, Giza, Egypt; d Department of Mechanical Engineering, Palestine Technical University – Kadoorie, Tulkarm, Palestine; e Department of Electrical Engineering, National Kaohsiung University of Science and Technology, Kaohsiung City, 807618, Taiwan; f Department of Electrical Engineering, Faculty of Engineering (Shoubra), Benha University, Cairo, 11629, Egypt; g Interdisciplinary Research Center for Sustainable Energy Systems (IRC-SES), King Fahd University of Petroleum and Minerals, Dhahran, Saudi Arabia; h Department of Energy and Process Engineering, Norwegian University of Science and Technology (NTNU), Trondheim, 7491, Norway

**Keywords:** Artificial intelligence (AI), Renewable energy, Microgrid, Energy storage, Supercapacitors

## Abstract

The field of electrical energy is moving through many rapid changes and continuous development, especially with the huge boom in the field of artificial intelligence (AI) and the presence of renewable energy systems (RESs). Simultaneously with this development, it was necessary to have energy storage systems such as super capacitors and batteries. This necessity increased with sudden changes in climate or loads that affect the output of generation systems such as solar and wind energy systems. The main goal of this study is to focus on supercapacitors as a future solution with high reliability. It studies all the various factors and operational modes such as the principle of operation, installation, types and modeling of supercapacitors. The study also focused on the employment of supercapacitors in diverse applications, especially in microgrids with their various systems including control and energy management strategies for these systems and investigating the integration between AI, supercapacitors and microgrids and the outcome of this integration. Besides, this research intends to a proposal for a hybrid energy storage systems (HESS) suitable for different loads, including solar cells as main source with supercapacitors and batteries. In addition, it proposes the energy management system (EMS) as well as appropriate control system, to solve the problems of utilities and microgrids and meet the power demand of loads for different operating conditions.

## Highlights

• Comprehensive review of micro-grids and challenges in energy management and stability.
• Examine the role of supercapacitors in improving the performance of micro-grid.
• Analyze developments in the technology of supercapacitors, including innovations in the material.
• Optimize incorporation approaches for supercapacitors, including hybrid and placement systems with batteries.
• Evaluate performance metrics such as lifecycle costs, energy efficiency and response time improvements.

## 1 Introduction

**Fig. 1:**
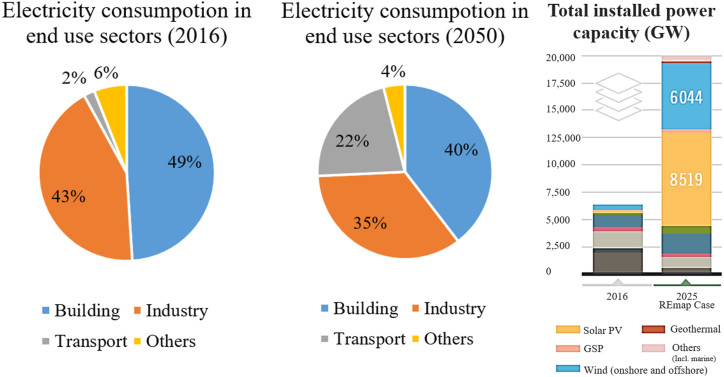
RE map case 2016–2050: Electricity and power generation installed capacity by fuel [4].

Renewable energy offers two main features that make it essentially ideal as a source of energy production [1]. It is inexhaustible and environmentally friendly as long as it does not produce waste materials and pollute [2,3]. Its importance has increased with the increasing tendency to integrate large quantities of distributed renewable energy into the electrical grid. According to analysis by (IRENA, 2019a), the proportion of global renewable energy generation is predicted to increase today from 25% to 86% in 2050 as shown in Fig. 1 [4]. The increase is robust for variable renewable energy technologies, especially wind power and solar (PV), increasing from 4.5% of power generation to almost 60% between 2015 and 2050 [4].

Some inconvenience, especially when using wind and solar energy sources, which are considered the most important sources of new and renewable energy, is due to high fluctuations, and continuous interruptions and this is because they depend on weather conditions and their behavior does not change only every hour but also seasonally [5]. Studies indicate that about 30%–40% of production in renewable energy systems (RESs) can be affected by weather fluctuations [6]. These results in the unpredictability of the primary energy source, which also causes difficulty in predicting the energy produced. This means the existence of maximum production during the period of minimum demand or generation surplus in congested parts of the electrical grid. This causes bottlenecks and overvoltage in some critical parts of the network [6,7]. In some areas, excess renewable energy production can lead to bottlenecks of up to 15% of total energy production, increasing the pressure on the grid. It is estimated that excess energy production can occur at peak times when production periods do not coincide with demand periods. Frequent outages caused by instability in renewable energy sources can cause power outages of up to 20% in some cases, affecting industrial and residential facilities [9,10]. However, with the rapid development of energy technologies, a priority for studies and research has been the need for energy storage to improve the energy quality and reliability of PV systems at the distribution level.

**Fig. 2:**
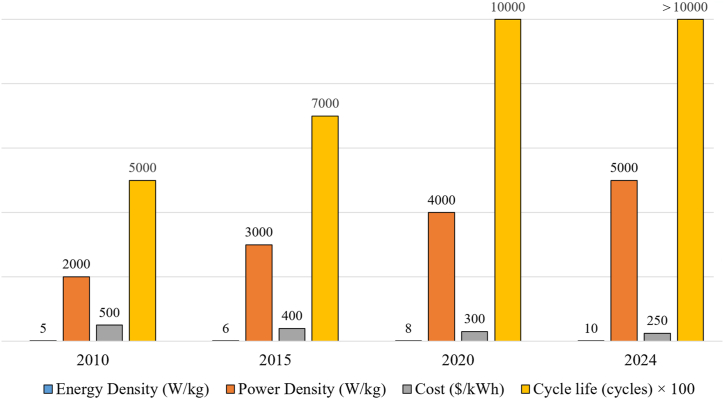
Supercapacitor performance metrics over time.

Effective energy storage would contribute significantly to achieving essential goals such as removing the high carbon levels associated with power generation, energy safety and reliability, and energy price stability [11]. Using energy storage systems (ESSs) can reduce voltage and frequency fluctuations by up to 50%, enhancing grid stability. Integrating ESSs with RESs can also increase grid reliability by up to 30% [12,13]. The great importance of supercapacitors (SC) in electrical ESSs is now apparent because of their very important characteristics, such as high power, longer life span, high response speed, and environmental friendliness [14]. It can be said that they combine the characteristics of traditional capacitors, batteries, and fuel cells (FCs) in terms of power and high energy [15]. The advantages of supercapacitors increase when they are combined in HESS with other storage elements such as batteries or FCs. These characteristics and advantages of supercapacitors which is demonstrated by the performance measures during the period from 2010 to 2024, as shown in Fig. 2 [16,17] make them suitable and very useful in their employment for some applications, which will be dealt with in subsequent parts of this review. Among the most important of them is the improvement of grid inertia. Network black start state, regulation of voltage and frequency, smoothing of renewables intermittency and management of energy.

Technological developments in the field of materials are considered one of the most important factors that have contributed to the significant improvement in the performance of supercapacitors. Among these developments is the development of advanced nanomaterials and carbon structures such as graphene, which play a major role in increasing the efficiency of energy storage and improving the ability of capacitors to respond quickly to load changes. Graphene, in particular, has superior electrical conductivity, allowing for the storage of larger amounts of charge compared to conventional materials used in capacitors [18]. For example, advances in the use of graphene have increased the ability of supercapacitors to store energy by up to 300% compared to conventional materials [19]. Also thanks to improvements in manufacturing processes and the reduction of toxic materials, the carbon footprint of the new supercapacitors is reduced by 40%–50% compared to the previous generation [20]. Moreover, recent research has been able to improve the designs of supercapacitors, leading to reduced energy losses and increased energy conversion efficiency. These technological developments have made supercapacitors suitable for use in a variety of applications, ranging from microgrids to large systems such as smart grids and electric transportation systems. Just as SC play an important role in microgrids that rely on traditional batteries, supercapacitors can take over the role of handling rapid and severe loads, relieving stress on batteries and extending their life.

**Fig. 3:**
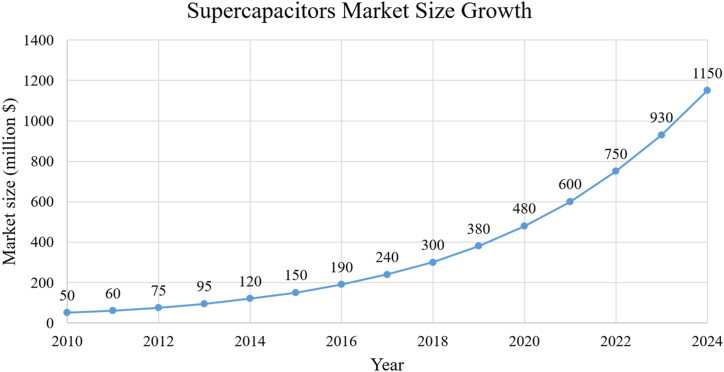
SC market size growth (2010–2024).

**Fig. 4:**
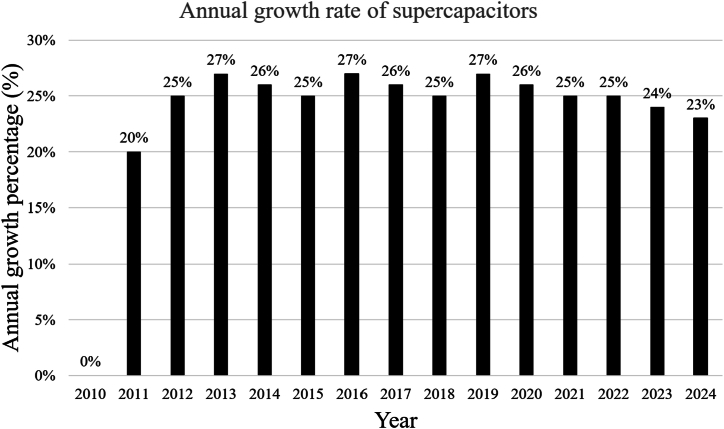
Supercapacitors annual growth rate (2010–2024).

**Fig. 5:**
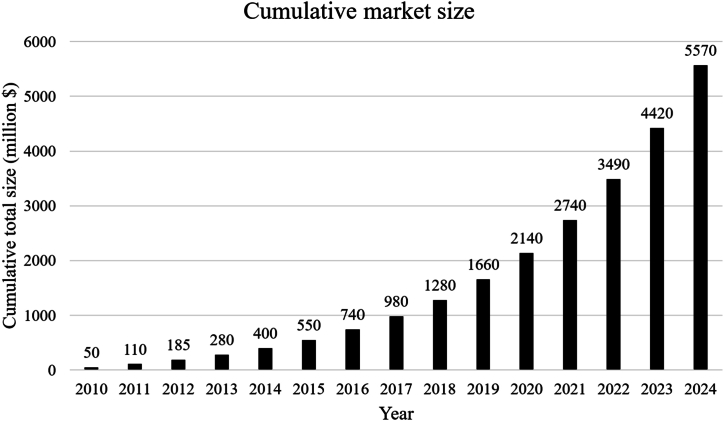
Cumulative market size chart (2010–2024).

With the spread of microgrids and their integration with renewable energy sources, whether the work is off grid or on grid, there has been an accelerated growth for SCs showing the growth from $50 million in 2010 to approximately $1150 million in 2024 as shown in Fig. 3 [21], an amazing growth rate, indicating the increasing demand for supercapacitor technologies due to their advanced energy storage capabilities and rapid response. The graph in Fig. 4 shows that market growth has been strong and steady, with annual growth rates ranging between 20% and 27% over the years with a slight gradual decline in the percentage growth as the market matures [22]. The drivers of this growth were the increasing use of renewable energy such as solar and wind energy in microgrids. These microgrids require efficient and rapid response storage solutions such as supercapacitors, which are one of the main factors behind this growth. The chart in Fig. 5 shows the cumulative growth of supercapacitor usage in microgrids, showing how the market has doubled in 14 years [23]. One of the most important future expectations is that with the continued global shift towards sustainability and clean energy, it is expected that reliance on supercapacitors in microgrids will continue, especially with new technological developments that increase their efficiency and reduce their cost.

Closely related to the topic of MGs, SCs and the development of RES, the world is currently experiencing a technological boom, especially in light of the spread of artificial intelligence (AI) applications in various fields. This has confirmed the importance of integration between AI, SCs, and MGs. This integration opens the door to a future full of innovations in the energy field. With the continuous improvement in AI technologies and the development of new materials for SCs, these systems are expected to become more efficient and effective in providing sustainable and safe energy solutions, and this is also what we will highlight in this review.

Given the importance of SCs in MGs and the different configurations of RESs and the launch of a new era of technology known as AI, this was the motivation for creating this review. There were many studies that contributed and addressed topics related to the current research topic. Therefore, Table 1 summarizes a comparison between the current study and a group of other studies that were addressed in the research. These introduction highlights several key points about the growing importance of renewable energy, the challenges it faces due to variability, and the role of energy storage systems (ESS), particularly supercapacitors (SC), where it addressed the following main points.

• Renewable Energy Growth.
• Challenges of Renewable Energy.
• Importance of Energy Storage.
• Supercapacitors (SCs) in Energy Storage.
• Technological Advances in Supercapacitors.
• Market Growth.
• Future Outlook.

**Table 1:** Summarizes a comparison between the current study and a group of other studies.

Ref. No	Year	Subject/Focus	Study Objective	Methodology Used	Key Findings	Practical Applications	Keywords
[1]	2021	Discussion of pros and cons of renewable energy sources	Assessing the feasibility of renewable energy	Literature review and critique	Clarifying challenges and opportunities	Promoting renewable energy use	Renewable energy, benefits
[4]	2020	Valuing energy storage in renewable energy context	Analyzing importance of energy storage	Economic modeling	Providing storage strategies	Enhancing grid performance	Energy storage, grid
[6]	2023	Impact of renewable energy variability on grid reliability	Studying the impact of variability	Simulation modeling	Assessing grid reliability	Improving management strategies	Renewable energy, reliability
[9]	2024	Energy storage solutions for renewable energy variability	Analyzing energy storage solutions	Field study	Offering effective storage strategies	Improving energy sustainability	Energy storage, renewable energy
[10]	2022	Managing wind and solar energy impacts on the grid	Providing management strategies	Practical analysis	Enhancing grid performance	Guiding policies	Wind energy, solar energy
[12]	2022	Impact of energy storage systems on high renewable penetration	Studying impact on electric systems	Data analysis	Providing clear results	Improving system performance	Energy storage, electric systems
[13]	2023	Challenges and opportunities in renewable energy integration	Assessing challenges	Case study analysis	Offering integration recommendations	Enhancing integration strategies	Renewable energy, integration
[15]	2020	Comprehensive review of supercapacitors and applications	Evaluating technology advancements	Literature review	Providing clear results	Offering recommendations for applications	Supercapacitors, applications
[17]	2021	Progress and challenges in supercapacitor performance	Providing options for performance enhancement	Practical analysis	Presenting clear results	Enhancing improvement strategies	Supercapacitors, performance
[18]	2020	Advances in supercapacitor technology and materials	Evaluating new materials	Case study analysis	Presenting recent findings	Enhancing new materials use	Supercapacitors, materials
[19]	2022	Analysis of the supercapacitor market	Studying market size	Market analysis	Providing market data	Guiding investments	Market, supercapacitors
[20]	2022	Advanced nanostructured materials for high-performance capacitors	Providing new options	Case study analysis	Enhancing performance	Offering recommendations for research	Electrochemical capacitors, nanostructured materials
[21]	2020	Market analysis	Analyzing market trends	Market research	Significant growth projected	Informing investment strategies	Market trends, supercapacitors
[35]	2021	Pseudocapacitive materials	Reviewing material developments	Literature review	Identifying key materials	Guiding material research	Pseudocapacitive, supercapacitors
[37]	2023	Transition metal oxides	Analyzing electrode materials	Literature review	Highlighting performance advantages	Informing research directions	Transition metal oxides, supercapacitors
[40]	2021	Fuel Cell Systems	Overview of market trends	Market analysis	Fuel cells gaining traction in energy storage	Informing energy policy	Fuel cells, energy storage
[44]	2021	Supercapacitor Technology	Evaluating applications	Review study	Supercapacitors essential for modern applications	Supporting future designs	Supercapacitors, applications
[45]	2023	Supercapacitors	Advances in technology	Review study	Significant advancements in materials	Informing material choices	Supercapacitors, materials
[46]	2022	Lithium-Ion Batteries	Evaluating material challenges	Review analysis	Materials issues hindering performance	Guiding future research	Lithium-ion, materials
[47]	2023	Flywheel Energy Storage	Evaluating technology	Review analysis	Flywheel technology advancing rapidly	Informing technology strategies	Flywheel, renewable energy
[48]	2023	Hydrogen Fuel Cells	Overview of current status	Review analysis	Hydrogen fuel cells show promise	Informing fuel cell deployment	Hydrogen, fuel cells
[49]	2020	Hybrid Systems	Evaluating hybrid systems	Technical study	Hybrid systems can enhance stability	Informing system design	Hybrid systems, PV
[74]	2022	Microgrid Systems	Evaluating integration strategies	Case study	Integration improves overall performance	Informing system designs	Supercapacitors, integration
[86]	2023	Power Distribution	Evaluating power distribution control	Technical study	Bidirectional converters improve distribution	Informing system designs	DC/DC converter, hybrid systems
[88]	2023	Passive HESS	Overview of passive systems	Comprehensive review	Passive systems show significant promise	Informing design practices	Passive HESS, energy storage
[89]	2023	Semi-Active HESS	Evaluating optimization methods	Technical study	Optimization improves semi-active systems	Supporting optimization strategies	Semi-Active HESS, optimization
[90]	2023	Fully Active HESS	Evaluating design strategies	Technical study	Design strategies enhance system performance	Informing design choices	Fully Active HESS, energy storage
[91]	2023	Multi-Port HESS	Evaluating multi-port systems	Technical study	Multi-port systems present unique challenges	Supporting system designs	Multi-Port HESS, energy storage
[94]	2023	AC Microgrids	Review design trends in AC microgrids	Literature Review	Highlights the transition from basic to advanced AC microgrid designs	Smart grid implementation	AC Microgrids, Design, Smart Grid
[95]	2023	DC Microgrids	Analyze advancements in DC microgrid technologies	Literature Review	Identifies key architectures and control strategies for DC microgrids	Integration of renewable sources	DC Microgrids, Architectures, Control
[96]	2023	Hybrid Microgrids	Explore integration strategies for hybrid AC/DC microgrids	Literature Review	Discusses potential pathways for hybrid microgrid integration	Enhanced grid resilience	Hybrid Microgrids, Grid Integration
[97]	2023	Community Microgrids	Investigate the role of community microgrids	Case Studies	Community microgrids can drive sustainable transitions	Local energy independence	Community Microgrids, Sustainable Energy
[98]	2023	Remote Microgrids	Examine design challenges and optimization techniques	Case Studies	Identifies key challenges and optimization strategies for remote microgrids	Remote energy solutions	Remote Microgrids, Optimization
[119]	2021	AI in Energy Management	Review AI-driven approaches in microgrid energy management	Case Studies	Discusses effectiveness of AI in managing energy in microgrids	Optimizes energy management strategies	AI, Energy Management, Microgrids
[121]	2021	AI and Energy Storage	Assess the integration of AI with energy storage systems	Experimental and Simulation	Enhances performance of energy storage through AI integration	Optimization of energy storage in microgrids	AI, Energy Storage, Microgrids
[122]	2021	Energy Storage and Renewables	Analyze costs and market trends for electricity storage	Market Analysis	Provides insights into market trends for energy storage	Supports decision-making in energy investments	Energy Storage, Renewables, Market Trends
[123]	2023	Supercapacitors Market	Forecast market trends for supercapacitors	Market Research	Projects growth in supercapacitors due to rising demand	Informs investment strategies in supercapacitors	Supercapacitors, Market Analysis, Forecast
Current study	2024	Supercapacitors and AI in microgrid	Review of the integration of supercapacitors with AI in microgrid management in different operating modes	Literature review	Proposed system for feeding a microgrid	Improving Micro-Grid Management	AI, renewable energy, micro grid, energy storage, supercapacitors

This review sets the foundation for further investigation into the evolving role of supercapacitors in the renewable energy landscape and energy storage systems. It also explores the potential for integrating emerging technologies like AI to optimize their use. However, can supercapacitors replace batteries entirely in microgrids? Or will hybrid systems be the most effective solution? It is also interesting to see what role supercapacitors will play in smart grids and electric vehicles in the future.

The literature review which addressed in current study are divided into categories according to the topics covered by these studies, and a critical analysis of previous studies on the use of SCs in ESSs is presented. It includes identifying the strengths, limitations, and challenges of these studies, in addition to the gaps in knowledge that need further research as follows:

Advantages and disadvantages of renewable energy sources (RES) are discussed in Ref. [1], while benefits of RES are explored in Refs. [2,3] for the economic and environmental benefits. In addition, the issue of variability in RES are introduced in details in Refs. [[6], [7], [8], [9], [10], [11], [12], [13]] with highlighting the challenge of wind and solar energy due to variation in availability. In Refs. [9,14,15], authors discuss various energy storage solutions with focusing on SCs. The technology of supercapacitors including details of supercapacitor technology, recent advancements, materials, and applications are depicted in Refs. [16,39]. The authors in Refs. [40,41,[46], [47], [48]] presents a comparison of storage solutions such as supercapacitors, batteries and flywheels. Moreover, in Refs. [49,[52], [53], [54], [55], [56], [57], [58], [59], [60],[72], [73], [74], [75], [76], [77], [78], [79], [80], [81]] discusses how supercapacitors can be used in microgrids, which are small localized power grids. The combination of supercapacitors with other storage technologies like batteries has been explored as in Refs. [72,[75], [76], [77],^79^]. Microgrid control strategies are delved into control strategies for managing power flow and optimizing energy storage use in microgrids [86,87,92,93,[99], [100], [101]]. Moreover, different types of hybrid energy storage systems are categorized based on their control complexity as given in Refs. [[88], [89], [90], [91]]. Authors in Refs. [[94], [95], [96], [97], [98]] discusses different types of microgrid configurations such as AC, DC, hybrid AC/DC, community and remote.

The strengths of previous studies could be summarized as the studies covered a wide range of applications, including RES, electric vehicles, electronic devices, and MGs. Besides, several studies have been conducted to develop new materials with higher performance for SCs, resulting in improved energy density and power density. Moreover, significant progress has been made in improving the performance of SCs in terms of cycle life, efficiency, and cost. Advanced simulation models have been developed to analyze the performance of ESSs based on SCs. In addition, the integration of SCs with different power systems, such as electrical grids and MGs, has been studied. However, the limitations and challenges for previous studies includes that most of studies often focus on laboratory performance of supercapacitors, and may not necessarily reflect performance in real operating conditions. Besides, there is a lack of studies that address the long-term performance of SCs in practical applications. Moreover, the cost of SCs is still high compared to batteries, which limits their deployment in some applications. More studies are needed to evaluate the life cycle of SCs and their environmental impact. Moreover, supercapacitors face challenges in thermal management, especially under harsh operating conditions.

The gap is that studies may need to test hybrid systems in real operating environments to obtain reliable data on performance and reliability. Besides, there is a need to develop new materials with higher energy density and power density, with longer cycle life and lower cost. Advanced control strategies need to be developed to improve the performance of energy storage systems based on supercapacitors. More accurate simulation models must be developed to predict the performance of supercapacitors under different operating conditions. Understanding the potential for scaling up the use of supercapacitors at the large power system level is still at an early stage. Moreover, a comprehensive life cycle cost analysis of supercapacitors needs to be conducted to identify optimal applications. Standardized testing standards necessity to be developed to reliably evaluate the performance of supercapacitors.

The main contributions of this paper could be summarized as follows:

• Compare for supercapacitors and other storage devices from perspective (nature, energy, power density, power delivered, storage time, benefits and drawbacks).
• Analyses the importance of supercapacitors in micro-grid systems.
• Discusses the integration of supercapacitors across various operating scenarios.
• Highlights the distinct role of supercapacitors in enhancing the stability and efficiency for micro-grid systems.
• Summarizes recent growths in technology of supercapacitor.
• Identifies potential areas and challenges for further research in management of micro-grid.
• Investigating the integration of AI, supercapacitors and microgrids.
• Statistical analysis of comparisons between supercapacitors and other storage systems.

## 2 Supercapacitors

This section presents the working and specifications of supercapacitors, its modelling, classifications and materials.

### 2.1 Working and specifications

**Fig. 6:**
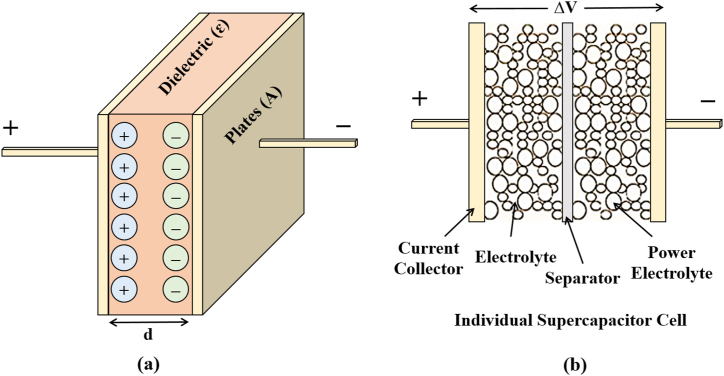
(a) Structure of an electrostatic capacitor, (b) Structure of supercapacitor.

Electrochemical capacitors differ from conventional capacitors in storing electrical charges by using a double layer and the absence of a dielectric material, where the negative and positive ionic charges of the electrolyte gather on the surface of the conductive electrodes to compensate for the opposite electronic charges on the electrode surfaces which is described in Refs. [[24], [25], [26]]. Hence, the bilayer capacitance arises at the interface between the electrodes and the electrolyte, according to Eq. (1). The description of the process is given in Fig. 6(a) and (b) and mentioned in Refs. [25,27,28].

(1) $$C=\frac{\varepsilon_{0}\times \varepsilon_{r}\times A\;}{d}$$

### 2.2 Modeling of supercapacitor

**Fig. 7:**
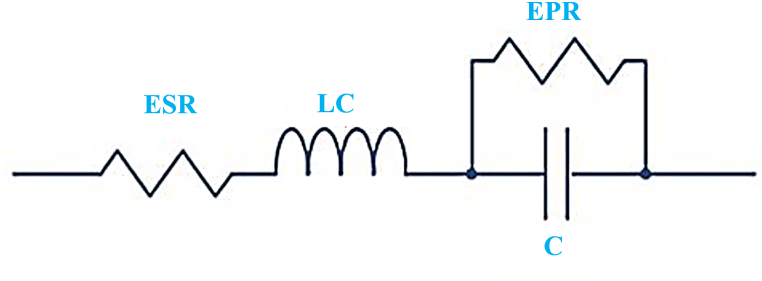
SC Electrical equivalent circuits model [29,30,32]**.**

The supercapacitor is represented by a first-order circuit in Fig. 7 with components including equivalent series resistance (ESR), series inductance (SI), and equivalent parallel resistance (EPR) along with a capacitor (C) [29,30]. The SC bank energy (Esc) is directly proportional to the change in terminal voltage and the capacitance as demonstrated in Eq. (2) [31]. Eqs. (3), (4), (5) are used to describe the ESR, R_sc−total_ and C_sc−total_ respectively. Moreover, the maximum discharge power of SC (Pmax) can also be represented by Eq. (6).

(2) $$E_{\text{sc}}=\frac{1}{2}\;C\;\left(\;V_{i}^{2}\;–\;V_{f}^{2}\;\right)$$

where, Vi is the initial voltage before start discharge (Volt) and Vf is the final voltage after start discharge (Volt)

(3) $$\text{ESR}=\frac{\Delta \text{Vd}}{\text{Id}}$$

(4) $$R_{\text{sc}-\text{total}}=\text{ns}\;\frac{\text{ESR}}{\text{np}}$$

Rsc−total is defined as the total resistance for SC system (Ω)

(5) $$C_{\text{sc}-\text{total}}=n_{p}\;\frac{C}{n_{s}}$$

C is the SC capacitance (F), and Csc−total is the total capacitance for SC system (F)

(6) $$\text{Pmax}=\frac{V_{\max}^{2}}{4\text{ESR}}$$

Pmax is the maximum dischargeable power, and Vmax is the SC maximum voltage.

### 2.3 Classification of supercapacitors

Supercapacitors store electrical energy depending on two types of capacitive nature such as classified as following:

• Electric double-layer (EDL) capacitance,
• Pseudo capacitance.

**Fig. 8:**
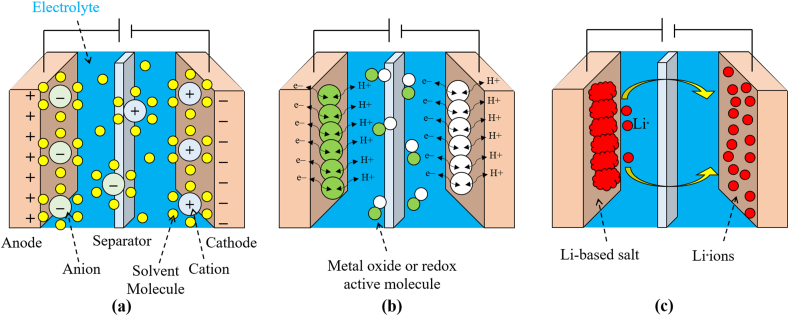
(a) Edlc, (b) PC and (c) HSC.

The charge storage process in EDLC is shown in Fig. 8 (a) at the electrode and electrolyte interfaces. As demonstrated in Fig. 8 (b), pseudo capacitors (PCs) use quick, reversible Faradaic redox processes to store charge and boost a SC's capacitance. As illustrated in Fig. 8 (c), the charges stored in (HSC) by coupling a pseudo capacitive or lithium-insertion electrode and a capacitive carbon electrode as explained in Refs. [15,25,33]. Table 2 and Table 3 compare the three basic types of supercapacitors such as EDLC, pseudo capacitors, and HSCs [15,34].

**Table 2:** Comparison between supercapacitor types (EDLC, PC and HSC).

Parameters	Electrochemical double-layer capacitor (EDLC)	Pseudo capacitors	Hybrid capacitor
Electrode material	Carbon	Metal Oxides (MOs) and Conducting polymers (CPs)	Both Carbon and MOs/CPs
Charge storage phenomenon	Non-Faradaic	Faradaic	Both Faradaic and non-Faradaic
Specific capacitance	Low	High	Intermediary
Specific energy	Low	High	High
Rate capability	Good	Low	Good
Cycling stability	Good	Low	Moderate

**Table 3:** A comparison summarizing the performance of the SC types [35,36].

Type	Energy Density (Wh/kg)	Power Density (W/kg)	Cycle Life (cycles)	Charge/Discharge Time	Typical Materials
EDLCs	5–10	10,000–20,000	>1,000,000	Seconds to minutes	Activated carbon, graphene, CNTs
PCs	10–50	1000–10,000	10,000–100,000	Seconds to minutes	Conducting polymers, metal oxides
HSCs	20–80	5000–15,000	10,000–100,000	Seconds to minutes	Lithium-ion capacitors, composite electrodes

### 2.4 Supercapacitor materials

#### 2.4.1 Electrode materials

The materials from which the electrodes of the supercapacitors are made are considered one of the most important factors affecting the value of the electrical capacitance. This led to the most widely used materials, such as carbon materials due to their high surface area, metal oxides that provide suitable options due to their high specific capacity and low resistance, and conductive polymers that are used as electrode materials [36]. In Table 4 comparison summarizing the different materials used in supercapacitors, along with their key properties and performance characteristics.

**Table 4:** A comparison summarizing the different materials used in SC [37].

Material	Specific Capacitance (F/g)	Energy Density (Wh/kg)	Power Density (W/kg)	Cycling Stability	Conductivity	Cost
Activated Carbon	100–250	5–7	10,000	>10,000 cycles	Moderate	Low
Graphene	200–550	8–15	20,000	>20,000 cycles	High	High
Carbon Nanotubes	150–200	10–20	30,000	>10,000 cycles	Very High	Moderate
MXenes	400–1500	10–30	30,000	>20,000 cycles	High	High
Conducting Polymers (e.g., Polyaniline, Polypyrrole)	300–500	1–5	1000–5000	<10,000 cycles	Moderate	Moderate
Metal Oxides (e.g., RuO₂, MnO₂, NiO)	300-1600	20–50	10,000–50,000	<5000 cycles	Low-Moderate	Very High
Hybrid Materials (e.g., Composite of Carbon and Metal Oxides)	300–600	10–30	15,000–30,000	>15,000 cycles	High	High

#### 2.4.2 Electrolytes

**Fig. 9:**
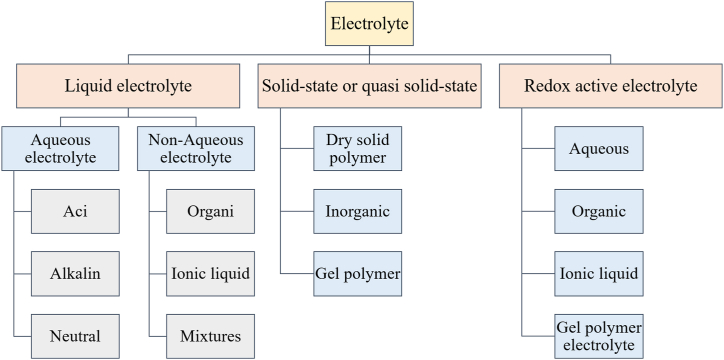
Classification of electrolytes for SCs applications.

Electrolytes are among the components that significantly impact supercapacitor performance. Electrolytes are categorized into organic liquids, ionic liquids, solid or semi-solid states, as well as redox-active electrolytes as illustrated in Fig. 9 [38].

#### 2.4.3 Separators

To reduce ESR, this separator must be highly porous and might be quite thin. During the early phases of SC development, natural materials like glass were employed as separators. The market for separators is now being led by developed polymer-based separators that are inexpensive, highly flexible, and porous as discussed in Refs. [25,40].

### 2.5 Supercapacitor V.S other storage devices

**Fig. 10:**
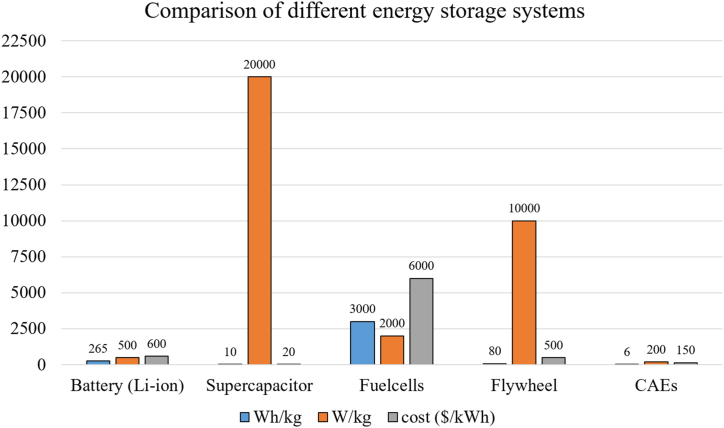
Comparison of different ESSs.

**Fig. 11:**
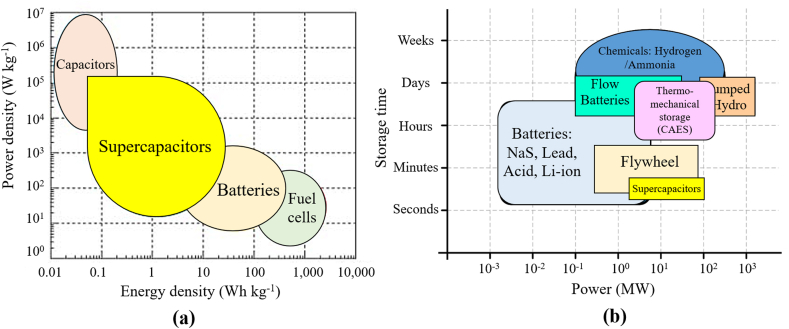
(a) Energy and power density,(b) Power delivered and Storage time for ESS [24,25,28,44,45].

The graph in Fig. 10 shows that fuel cells have the highest energy density, ranging from 1000–3000 Wh/kg, while compressed air storage and supercapacitors come in last, with energy densities of only 5–10 Wh/kg. It also shows that supercapacitors lead with power densities of 10,000–20,000 W/kg, followed by flywheels with power densities of 1000–10,000 W/kg, making them best for applications that require fast power. The graph also shows that fuel cells are the most expensive, ranging from $4000–6000 per kWh, while supercapacitors are relatively cheap at $10–20 per kWh [[41], [42], [43]]. As shown in Fig. 11(a) and (b) a quick comparison between different ESSs in terms of storage time, power density and power delivered. The low specific energy and high-power density are characteristics of SC. A comparison of SC with other energy storage devices are shown in Table 5, Table 6.

**Table 5:** Comparing supercapacitors with other energy storage devices [[46], [47], [48], [49]].

Factor	Supercapacitors	Lithium-Ion Batteries	Flywheels	Fuel Cells
Energy Density	Low (1–10 Wh/kg)	High (100–265 Wh/kg)	Medium (20–100 Wh/kg)	Very High (800–1000 Wh/kg)
Power Density	Very High (10,000 W/kg)	Moderate (100–1000 W/kg)	Low (1000–5000 W/kg)	Low (20–1000 W/kg)
Cycle Life	Extremely High (>1 million cycles)	Moderate (500–3000 cycles)	High (20,000+ cycles)	High (>5000 cycles)
Charge/Discharge Time	Very Fast (seconds)	Moderate (1–2 h)	Very Fast (seconds to minutes)	Slow (minutes to hours)
Efficiency	High (>95%)	High (85–95%)	High (90–95%)	Moderate (40–60%)
Cost	High per kWh but low per cycle	Decreasing but still high per kWh	High initial cost, low operating cost	High initial and operating costs
Temperature Sensitivity	Low	Moderate	Moderate	High
Environmental Impact	Low (mainly carbon-based materials)	High (reliance on mining and complex recycling)	Moderate (mechanical wear and tear, materials)	Low when using hydrogen, but depends on fuel source
Application	Power stabilization, quick bursts of energy	Portable electronics, electric vehicles	Grid storage, UPS (Uninterruptible Power Supply)	Backup power, remote applications
Scalability	Limited	High	Moderate	High

**Table 6:** Benefits and drawbacks of Supercapacitors V.S the Batteries and flywheels [[45], [46], [47], [48], [49], [50]].

ESS Technology	Advantages	Drawbacks
Batteries	- Low self-discharge - Narrow range of voltage variation in operation - High energy density - Low installation cost	- Accelerated aging with large power pulses - Low recyclability of materials Reduced operating temperature range - Low power density - Need Battery management system
Supercapacitors	- High power density - Wide operating temperature range Ageing not dependent on the duty cycle - More stable efficiency throughout the operating range - Quick charging/discharging - Does not blow up in case of accidental direct short connection - Stops accepting energy when it becomes fully charged - Internal ESR is extremely small (≈0.01 Ω) - Environmentally safe and no gas emissions - Extended lifetime and long shelf life (4–5 year)	- Wide voltage variation in operation Power converter required to operate - Voltage balancing system between cells required - Low energy density - Higher cost (€/kWh) - Very high self-discharge rate (≈1–2 days) - Highest dielectric absorption
Flywheels	- High energy density - Power and Energy decoupling Less ageing than batteries and supercapacitors - Very wide operating temperature range	- High self-discharge - High installation cost - Lower efficiency than batteries and SCs - Power converter required

## 3 SCs facing energy stability challenges

Supercapacitors provide significant support in solving energy storage problems and challenges that are exacerbated by the variability of renewable energy sources, which directly impact grid stability. The challenges of supercapacitors are to overcome the rapid response to fluctuations, short term storage, high efficiency and durability, integration with batteries, regulating grid frequency, and reliance of fossil fuels [50,51]. Renewable energy sources such as wind and solar are subject to constant climate change, and produce energy in an unpredictable manner. Supercapacitors have the ability to provide rapid bursts of energy due to their high energy density and rapid charge and discharge capabilities. This makes them ideal for grid stabilization by responding quickly to sudden changes in energy production or demand, mitigating fluctuations and preventing potential outages or instability.

SCs are well-suited for short-term energy storage, which is critical for managing intermittent RESs. While conventional batteries are better for long-term storage, SCs can bridge the gap between generation and demand by providing rapid energy storage and release during brief periods of excess power or sudden power shortages. This ability helps balance supply and demand more effectively on a minute-by-minute basis, enhancing grid reliability. SCs have a long cycle life and can operate without significant degradation while withstanding a large number of charge and discharge cycles. This durability is essential for energy storage systems that need to operate continuously to stabilize the grid. Their efficiency in storing and releasing energy means that more renewable energy can be stored and utilized with minimal loss, increasing the overall effectiveness of renewable integration into the power grid.

Supercapacitors enhance traditional energy storage solutions such as lithium-ion batteries. While batteries are suited for long-term energy storage, supercapacitors efficiently handle rapid power surges and fast charging cycles. Integrating supercapacitors with batteries in hybrid energy storage systems maximizes the strengths of both technologies, thus improving energy storage for both short-term stability and long-term storage needs. One of the main challenges for RESs is to maintain grid frequency within a certain range, to ensure stable operation. Supercapacitors help regulate frequency by releasing or absorbing energy quickly to balance power fluctuations and maintain grid stability and help maintain a steady supply of power, even during times of rapid change in renewable energy production [52]. SCs support RES in a way that helps reduce reliance on traditional fossil fuel-based backup power sources. These used to operate at peak times, to step in during periods of low renewable energy production. This could lead to a cleaner, more sustainable energy grid.

## 4 Applications of supercapacitors

Highly efficient ESS may help several electrical devices and applications, including vehicles, MG, green power production, mobile devices, data storage devices, and many more. The supercapacitor can be integrated into the system to implement ESS [[51], [52], [53]].

### 4.1 Black start

The ability to restart the network in an emergency, therefore storage is crucial since it supplies the energy required to restart the system. Because there are fewer controllable variables (micro-sources, loads, and switches) in distributed generation MGs than in traditional power systems, the restoration procedure is significantly easier. When a MG is linked to the grid, the ESS maintains system stability while handling its transient requirements [56]. It is often modeled as a pair of constant DC-VSs and a (VSI). To prevent any substantial voltage and frequency fluctuation, the ESS's capacity should be considered when determining the amount of power to be connected. As a result, BESS and SCSS provide supplementary power and transient power, respectively, during the black-start procedure [57].

### 4.2 Micro grid micro generation

The supercapacitor is used in the micro source system connected to MG as an energy storage device. Such a system has zero or extremely little inertia. Any immediate imbalance in active power can compromise the system's stability. Energy storage devices are connected to the grid using power electronics devices to provide steady functioning in the event of any mismatch [58].

### 4.3 In transmission lines

Supercapacitor technology has been elevated to the level of power transmission. Supercapacitor energy storage is a technical reality for high-power applications in the grid, improving the quality of power, stabilizing frequency and voltage, optimizing power transmission limits to renewable energy, and supporting distributed generation. The addition of an ESS can increase the stability of the transmission system because it is predictable that integrating transmission supercapacitor (TSCAP) technology will result in a much faster response time and lower costs compared to other storage technologies and traditional generation methods for every KW/KVA [57,58].

### 4.4 Smoothing of renewables intermittency

The output power from a renewable source, like solar, can vary by over 50% second by second as a result of changes in the weather. In a hurry to stabilize output power, SCs can inject power into MG or the main grid.

### 4.5 SC- UPS

It is possible to use supercapacitors without any batteries in designing a backup system to operate critical loads that need a rapid passage of some seconds, and this will be useful [59].

### 4.6 In a wind turbine

Batteries are noted for performing well at low temperatures and having a lifespan even in harsh environments. However, they must be replaced repeatedly throughout their lives in wind power plants. These plant are to deliver power bursts in the second range for blade of rotor changes across several hundreds of thousands of cycles. Supercapacitors provide a simple, solid-state, and extremely reliable solution to quickly satisfy power supply and demand since they have no moving components. Additionally, they have a large working range of temperatures, a long lifespan, flexible management, a reduced size of the system, and are both costs efficient, and extremely dependable [[57], [58], [59]].

### 4.7 The real-world impact of SCs in MGs (case studies)

The use of SCs in MGs at scales around the world offers the potential to enhance power stability, manage fluctuations in renewable energy production, and provide power with rapid response during peak loads and any changes in climate [61]. The following case studies illustrate how SCs are being integrated into MG systems to support grid reliability and sustainability around the world, from Europe and the United States to Asia and Australia as presented in Table 7. The combination of SCs with other storage technologies can also provide an effective solution to the stability and resilience of power systems, helping power systems become more sustainable [49].

**Table 7:** Case studies illustrate impact of SCs in MGs.

Location	Description	Application	Results	Ref.
UCSD Microgrid Project	UCSD has built a microgrid that relies on renewable energy to reduce power consumption from the main grid. This grid uses supercapacitors to enhance grid stability and improve grid efficiency.	Supercapacitors were used to store excess power generated by solar panels and wind farms and provide it during periods of high demand.	Supercapacitors helped maintain voltage stability and power balance within the grid, reducing the need for backup power generators and improving overall grid responsiveness.	[62]
South Korea Microgrid Project	South Korea has implemented microgrids in several locations to provide energy efficiently. One such project is a solar microgrid in Jindo City.	The grid used supercapacitors to provide backup power and distribute it when solar power production fluctuations occur	Supercapacitors helped reduce gaps in power supply and enhanced the grid's ability to absorb production fluctuations, helping to reduce power losses and increase system efficiency.	[63]
Microgrid in San Jose, California	In San Jose, a microgrid was developed that used solar energy and energy storage using supercapacitors. This grid was intended to support residential and commercial areas in the event of power outages	Supercapacitors were used to store short-term energy and quickly regulate power distribution during peak periods	This solution reduced energy costs and increased reliability due to the faster response times of supercapacitors compared to conventional batteries.	[64]

Microgrid Project in Cape Town, South Africa	The City of Cape Town developed a microgrid to improve the response to renewable energy and reduce reliance on the main grid	Supercapacitors were combined with energy storage systems to store solar energy generated during the day and use it during peak hours	Supercapacitors helped reduce power losses and increase grid stability, which improved system response in high demand situations and reduced the need for backup generators.	[65]
Microgrid Project in Germany (E.ON)	The German company E.ON implemented a microgrid based on renewable energy in a rural area. This network was designed to provide stable power to a small town that relies on wind and solar energy.	Supercapacitors were used to improve the grid's response to sudden changes in production from renewable energy sources.	The use of supercapacitors improved the grid's voltage stability and energy balance. It also helped reduce the need for large batteries, which reduced costs.	[66]
China Microgrid Project (Xiong'an New Area)	Xiong'an New Area is a new city under development in China, aiming to be a model for sustainable smart cities. A microgrid was implemented to meet energy needs with advanced storage technologies	Supercapacitors were integrated as part of an energy storage system used to support the grid during peak periods and fluctuations in production.	Supercapacitors helped reduce voltage fluctuations and enhanced the grid's ability to handle intermittent renewable energy sources. The study also showed that this technology contributed to improved energy efficiency and grid stability.	[67]
New Zealand Microgrid Project (Rotorua Microgrid):	A microgrid in the city of Rotorua to provide sustainable energy to local areas. This grid relies on renewable energy sources such as solar and geothermal energy	Supercapacitors were used to store renewable energy and provide it during times when the grid requires additional power.	Supercapacitors contributed to enhancing grid stability and reducing energy losses. The study also showed that the use of this technology led to reduced operational costs due to improved operational efficiency.	[68]
Broken Hill Microgrid Project in Australia	A microgrid was developed in the Australian city of Broken Hill to improve the sustainability of the local electricity system using solar energy and energy storage.	Supercapacitors were part of the system to support the grid in emergency situations, providing instant power to maintain voltage stability.	Supercapacitors contributed to reducing reliance on conventional power from the main grid, which led to reduced energy losses and improved grid performance.	[69]
Fukushima Microgrid Project in Japan	After the Fukushima disaster in 2011, a microgrid project was implemented to provide reliable and sustainable power to the affected areas	Supercapacitors were used as part of energy storage solutions to support the grid during periods of need.	Studies have shown that supercapacitors contributed to improving grid stability and efficiency, increasing reliance on renewable energy and reducing reliance on fossil fuels	[70]
Tesla's Microgrid in Remote Areas	Tesla has implemented microgrid projects in remote and off-grid communities to supply power using renewable energy and battery storage	Although lithium-ion batteries are often at the core of Tesla's microgrids, supercapacitors have been used in hybrid systems for balancing power distribution and reducing the wear on batteries by handling peak load demands.	In these systems, supercapacitors are beneficial for extending battery life by taking on the rapid cycling tasks that would otherwise put strain on the batteries. This allows for more efficient energy usage, cost savings, and increased reliability in regions that are prone to power outages	[71]

From the previous case studies (see Table 7), various ideas can be drawn that illustrate the importance of supercapacitors in microgrids. These can be summarized as rapid response, cost and efficiency issues, and multiple applications. Supercapacitors provide instant power to improve grid stability during peaks or sudden fluctuations in output. Although supercapacitors are not as cheap as conventional batteries, their long life and high energy storage efficiency make them a viable long-term option. They can be used to support microgrids around the world, especially in areas that rely heavily on renewable energy.

## 5 Supercapacitors integration with microgrid

A potential next-generation energy network is the MG. It includes electrical energy storage components and electrical power-producing units, including solar cells, wind turbines, FCs, and microturbines. This would typically be a supercapacitor and battery. The ability of the MGs to interconnect with the power grid allows them to provide or demand power from it. The smart grid is the name of this set-up [51,60]. In addition to what was shown above for more applications of SCs, they have a strong impact on the efficiency of MGs. This impact can be summarized in Table 8 [61,62].

**Table 8:** Empact of SC on efficiency of MG.

Feature	Before Supercapacitors	After Supercapacitors	Improvement (%)
Voltage Stability	Low	High	30%
Frequency Regulation	Poor	Excellent	40%
Power Quality	Moderate	High	35%
System Reliability	Average	High	25%
Energy Storage Cost	High	Moderate	20%

### 5.1 Supercapacitors in (HESSs)

**Fig. 12:**
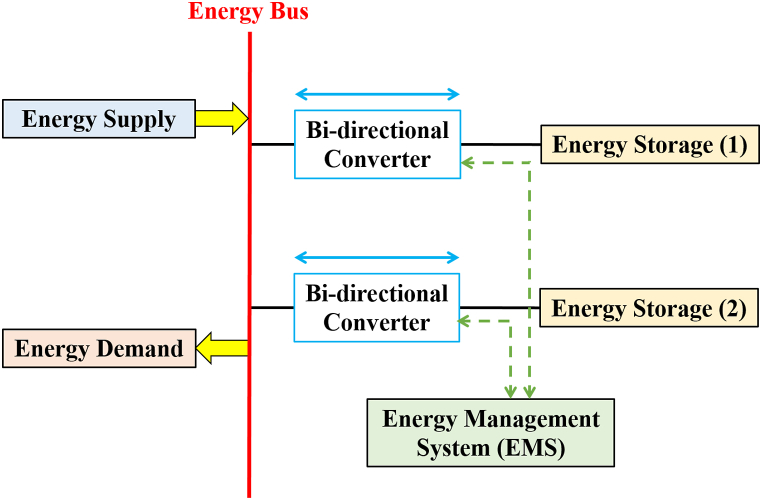
Basic structure of a HESS.

When using supercapacitors in electrical ESSs, they offer some advantages over other storage devices. This was agreed upon by researchers [60,[63], [64], [65], [66]]. Fig. 12 shows the basic structure of the HESS, which is characterized by the following [79].

• Lower overall investment costs compared to a single storage system (ES2 just needs to meet average power demand owing to the decoupling of energy and power).
• An improvement in overall system efficiency (due to ES2 dynamic loss reduction and ES2 running at optimal, high-efficiency operating points)
• Extension of storage and system lifespan (ES2's dynamic stress is reduced and its functioning is optimized)

#### 5.1.1 Battery- supercapacitor hybrid storage (BSHS)

**Fig. 13:**
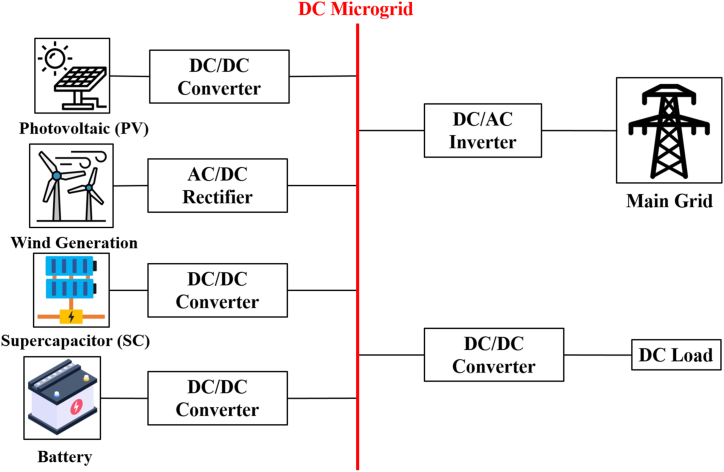
Shows a BSHS-configuration applied in microgrids.

While any generator may be integrated to produce electrical power, any collection of supercapacitors or batteries can likewise be used as electrical energy storage units [80]. The Micro-Grid's BSHS is an integration between batteries and supercapacitors. The battery serves as the primary energy storage component because of its increased energy density. A (BSHS) configuration for short- and medium-term coverage is shown in Fig. 13. Here, a supercapacitor serves as "high-power" storage, protecting against peaks, transients, and sudden changes in power. Some advantages of this composition include:

• Unbalanced load and harmonics: Battery and SC working together enhance MG performance under an imbalanced load. This results in quick and precise voltage control. Under unbalanced load situations [81].
• Reduce battery stress while preserving a high standard of power quality and dependability [72].

#### 5.1.2 PEM fuel cell - supercapacitor hybrid storage (FSHS)

**Fig. 14:**
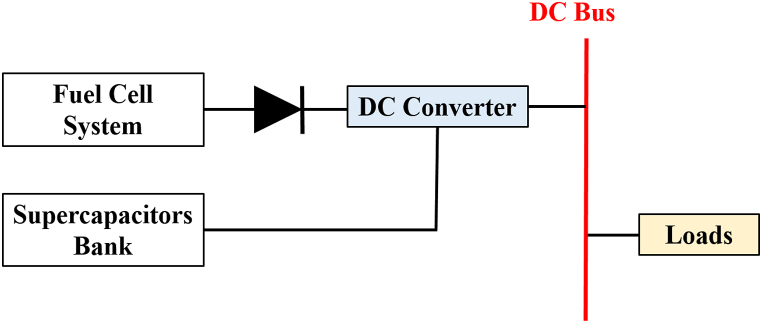
FC- SC hybrid system.

The FC power plant's power output could not be sufficient to fulfil sustained load needs, especially during times of high demand or transient occurrences. The combination of FC with SC offers the potential for improved energy efficiency, lower FC technology costs, and more efficient fuel use [31]. The SC and FC power systems can be integrated using a variety of topologies [70,71]. For instance, the integration of the SC system is possible via series and parallel connections with or without a power electronic converter. As seen in Fig. 14. To stop reverse current from supercapacitors flowing into FCs, a power diode is utilized [31].

### 5.2 Classification of the HESSs topologies

**Fig. 15:**
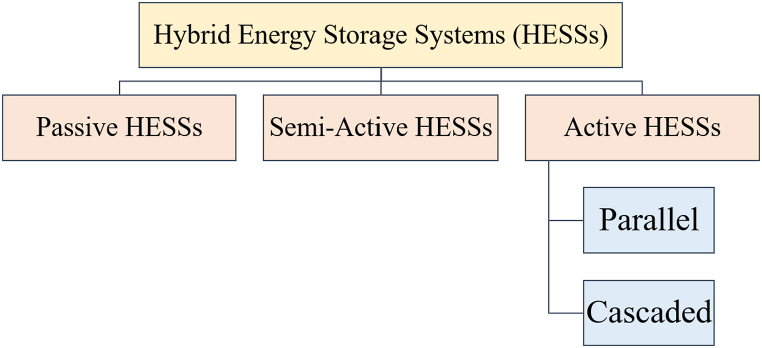
Taxonomy of the battery-supercapacitor HESS topologies.

**Fig. 16:**
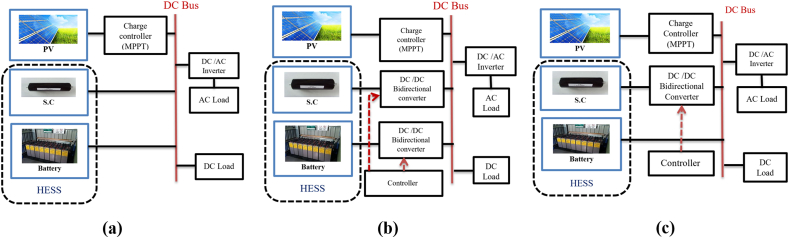
Supercapacitors in HESS: (a) Passive, (b) Active, (c) Semi-active (HESS).

**Fig. 17:**
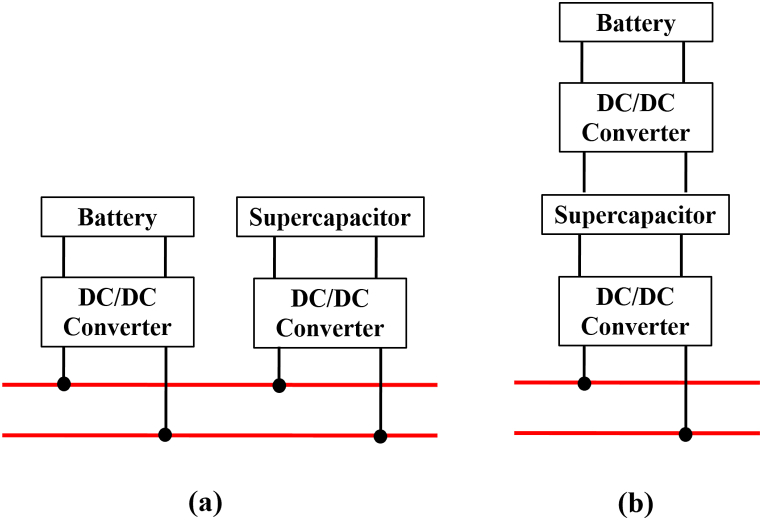
(a) Parallel, (b) Cascaded active HESS topology.

**Fig. 18:**
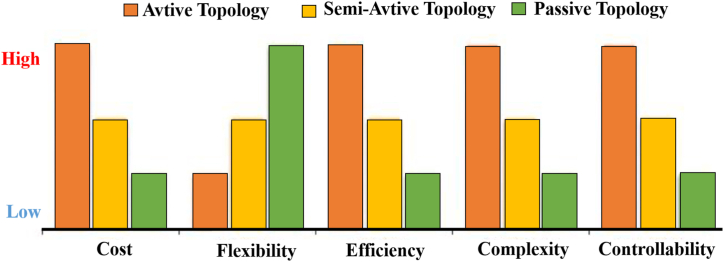
Evaluation of various (HESS) topologies.

The HESSs are classified in Fig. 15. This was agreed upon by the researchers [60,[72], [73], [74]] passive, active, and semi-active topologies, based on the linking between storage units and DC bus. If each storage unit and DC buses are directly connected, this configuration, known as passive HESS, as seen in Fig. 16 (a) but If each storage unit and DC bus are connected by a bi-directional DC/DC converter, this configuration is called active HESS, as shown in Fig. 16 (b). Semi-active storage is a one storage device that is connected to the DC bus by a bi-directional DC-DC converter, as demonstrated in Fig.16 (c) [50,60,75]. In addition, there are two methods in which active topology can be implemented, which are Parallel Cascaded active HESS topology as shown in Fig. 17(a) and (b). An MPPT charge controller in this setup regulates PV power, which powers the DC-MG. The bi-directional converters are provided to store energy from solar panels in batteries and supercapacitors in one way and to power the loads in another way using batteries and SCs [49]. The HESS topologies are compared from several operational perspectives in Fig. 18 [69].

A comparison of different HESS topologies is shown in Table 9, summarizing key performance metrics like energy efficiency, power density, energy density, cost, complexity, and more. This comparison is based on the most recent advancements in HESS technologies.

**Table 9:** A comparison of different (HESS) topologies [[76], [77], [78], [79]].

Metric	Passive HESS	Semi-Active HESS	Fully Active HESS	Multi-Port HESS
Energy Efficiency (%)	85–90	90–95	92–98	90–97
Power Density (W/kg)	500–1000	1000–1500	1500–3000	1200–2500
Energy Density (Wh/kg)	50–100	100–200	150–300	100–250
Cost ($/kWh)	Low (200–400)	Moderate (300–500)	High (400–700)	Very High (500–800)
Control Complexity	Low	Moderate	High	Very High
Scalability	High	Moderate	Low to Moderate	Low
Flexibility	Low	High (due to simplicity)	High	Very High
Reliability	Low (due to simplicity)	Moderate to High	High (due to redundancy)	High (depends on configuration)
Maintenance Requirement	Low	Moderate	High	High
Application	Small-scale systems (e.g., residential)	Medium-scale systems (e.g., commercial buildings)	Large-scale systems (e.g., industrial)	Specialized applications (e.g., advanced smart grids, military)

### 5.3 Micro-grid with HESS based on AC, DC and hybrid AC–DC bus

In a BSHSs, the two energy storage units can be connected to both (AC or DC) buses as mentioned in Refs. [60,80,81]. Table 10 represented comparative summary for different types of microgrids, covering aspects like size, control strategy, energy sources, application, and more. This information is aligned with current technological trends.

**Table 10:** Comparative summary for different types of microgrids [[82], [83], [84], [85], [86]].

Factor	AC Microgrid	DC Microgrid	Hybrid AC/DC Microgrid	Community Microgrid	Remote/Isolated Microgrid
Size/Scale	Medium to Large	Small to Medium	Medium to Large	Medium to Large	Small to Medium
Control Strategy	Complex (synchronization, frequency control)	Simpler (voltage regulation, fewer converters)	Complex (dual control strategies for AC/DC interfaces)	Complex (coordinated control for diverse sources)	Simplified (due to isolated operation)
Energy Sources	Typically, mixed (solar, wind, diesel, etc.)	Solar PV, batteries, fuel cells	Mixed (solar, wind, batteries, diesel, etc.)	Solar, wind, batteries, local generation	Diesel, solar PV, batteries
Efficiency	Moderate (depends on conversion losses)	High (fewer conversion steps)	High (optimizes both AC and DC loads)	Moderate to High	High
Grid Connection	Connected or islanded	Typically isolated, but can be connected	Can operate in both connected and islanded modes	Typically connected, but can island during outages	Typically islanded, may connect in emergencies
Reliability	High (due to established technology and standards)	High (simpler architecture, fewer points of failure)	Very High (redundancy in both AC and DC)	High (designed for community needs and resilience)	High (critical for isolated locations)
Cost	Moderate to High (due to control systems and converters)	Lower (fewer components, simpler control)	High (requires advanced control and interfacing)	High (community scale, diverse resources)	High (logistics and specialized equipment)
Scalability	High (well-established standards)	Moderate to High	High (can easily expand both AC and DC)	Moderate (depends on community growth)	Low to Moderate
Environmental Impact	Moderate (depends on energy mix and grid dependence)	Low (renewables-dominated, less infrastructure)	Low (optimized energy use, reduced losses)	Low (focus on sustainability and local generation)	Low to Moderate (depends on source mix)
Application	Industrial parks, campuses, military bases	Data centers, small commercial buildings, EV charging	Smart grids, large campuses, advanced industrial sites	Communities, neighborhoods	Remote villages, islands, mining operations
Complexity	High	Low to Moderate	High (dual system management)	High (coordinating community needs)	Moderate (simplified, resource-intensive)

#### 5.3.1 AC-coupled micro-grid

**Fig. 19:**
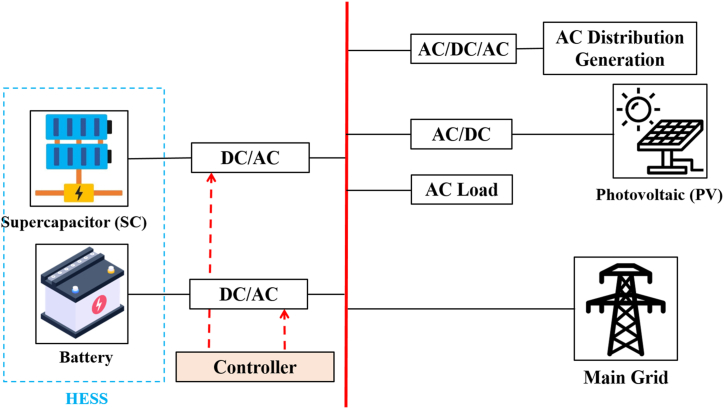
Ac coupled micro-grid.

**Fig. 20:**
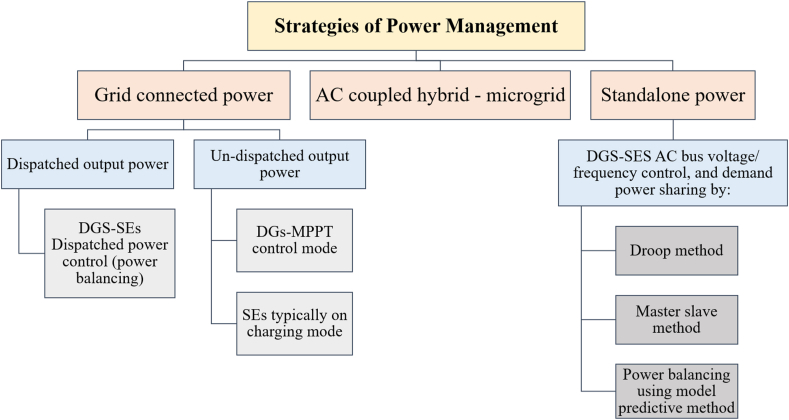
Power management strategies for AC coupled HMG.

By using interface converters, different distributed generators (DGs) and storage energy (SEs) may be connected to the shared AC bus in an AC-coupled microgrid (see Fig. 19). To enable bidirectional power flow, the bidirectional converters are crucial for SEs. The strategy of control and management of power plan Focus areas in AC-coupled systems, particularly in standalone operating mode, include (generation or consumption) of power balancing and AC bus (frequency and voltage) management. Moreover, Fig. 20 demonstrates different strategies of power flow management in AC microgrids depending on the nature of the configuration and connection with or without the public grid. Due to its straightforward design and straightforward control and power management strategy, the AC-coupled microgrid is now the most common form. SEs and DGs can be seen as shunt AC-VSs or CSs in this configuration [99].

#### 5.3.2 MG using DC-coupled technology

**Fig. 21:**
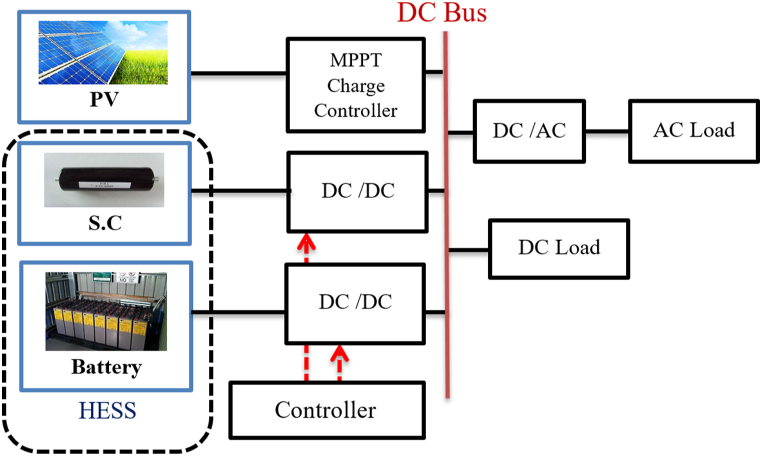
DC Coupled micro-grid.

The common DC bus in this topology connects DGs and SEs. This can be employed when the main units of power generation in the (MG) are DC power sources. It should be noted that both the SEs/DGs are linked to the DC bus, and that variable frequency AC loads like modifiable speed motors may also be linked to the DC bus using a DC/AC converter (avoiding the need for an extra AC/DC conversion for AC bus connection), as shown in Fig. 21. The common DC bus is the most popular option in standalone MGs for several reasons listed in Refs. [88,[89], [100]].

• The majority of ESS components and renewable energy sources run on DC voltage. Keeping a DC bus reduces the requirement for a power converter [90,91].
• Because DC buses don't need to be synchronized, the complexity of the entire system is considerably reduced. This was agreed upon by the researchers [60,75,92,93].
• The DC connection also makes it possible to employ DC loads, which have a number of advantages including lower power loss and voltage drop, greater efficiency, and lower total cost [[101], [102], [103]]. Therefore, compared to analogous AC bus systems, DC coupling is more cost-effective and efficient [94,95].

**Fig. 22:**
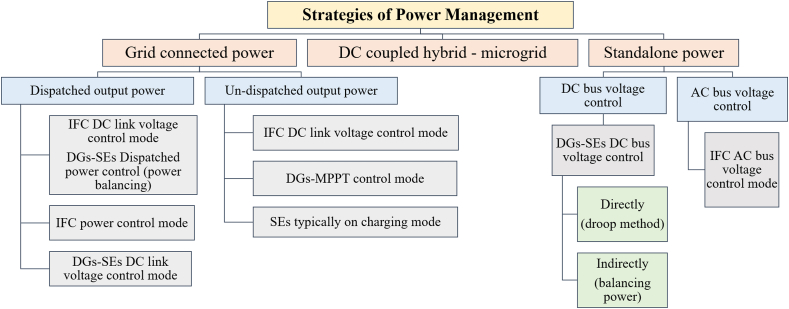
Strategies of power management for DC coupled HMG.

The power management goals techniques in HMG based on DC-coupled include the control of the DC link voltage, power balancing between generation and demand, and the control of the AC link voltage and frequency (particularly in standalone mode). The outline of the control and power management strategies of an HMG based on a DC-coupled system is presented in Fig. 22. The approaches to power management may also be separated into on-grid operation and off-grid operation, with the former having dispatched power mode and the latter having un-dispatched power mode [99].

#### 5.3.3 Hybrid micro-grid based on AC-DC-coupled

**Fig. 23:**
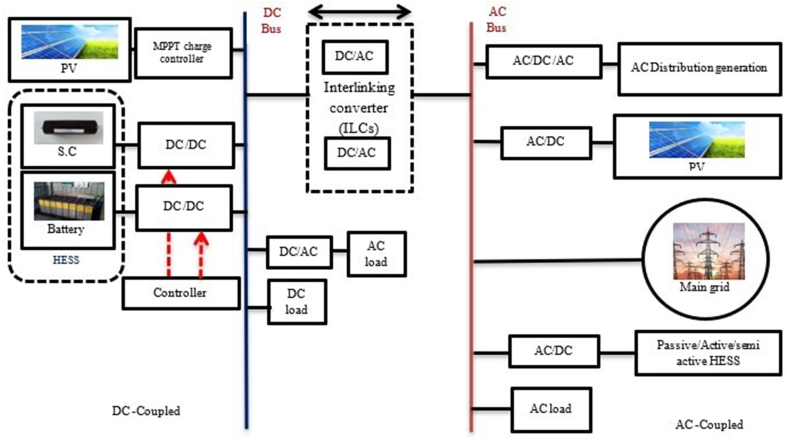
HMG based on AC–DC coupled.

**Fig. 24:**
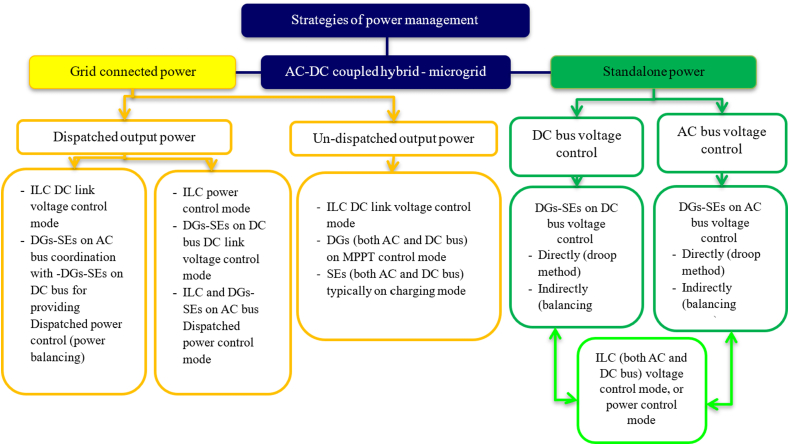
Strategies for managing power in HMGs based on AC-DC-coupled.

As demonstrated in Fig. 23 the (AC-DC) HMG is employed to satisfy the demands of AC and DC power producers and ESSs for more flexibility. Additionally, DC/AC and DC/DC converters offer a great degree of power flow controllability [80,87]. Fig. 23 illustrates the construction of an HMG with an AC-DC coupling. As seen in these configurations, DC buses are connected to AC buses by interlinking converters (ILCs), which both consist of (DGs/SEs). Unlike a DC-coupled system, which also comprises (DGs/SEs) on an AC bus, this topology necessitates more coordination between the (DC) and (AC) subsystems in order to regulate power and voltage. So as to connect the AC and DC buses with greater capacity and dependability, parallel ILCs are also necessary. This topology has increased overall efficiency and lowered costs. The control methods and energy management strategies for the power balancing and control of voltage in (DC-AC) subsystems should be considered. Fig. 24 describe the strategies for the management of power for a HMG that uses an (AC-DC) coupling [[99], [104], [105]].

It is possible to summarize the comparison between the types of MGs, which is shown in Table 11 [[106], [107], [108], [109]].

**Table 11:** Showing a summary of a statistical comparison between AC, DC and HMG.

Feature	AC Microgrid	DC Microgrid	Hybrid Microgrid
Efficiency%	85%–90%	95%–98%	90%–95%
Cost ($/kw)	500–800	700–1000	800–1200
Annual maintenance cost (% of initial cost)	5%–10%	10%–15%	12%–18%
Renewable Energy Compatibility	Medium	High	Very High
System flexibility	Low	High	Very High
Energy loss rate due to conversions (%)	8%–12%	2%–5%	5%–8%
Easy integration	Easy with traditional systems	Difficult with existing systems	Depends on the design

### 5.4 Recent advancements and practical implementation challenges

#### 5.4.1 For SCs in HESSs

Despite the advantages offered by supercapacitors in various applications, recent advances in supercapacitor technology, such as the development of advanced electrode materials and electrolytes, have led to significant improvements in their performance. Their use faces some challenges that prevent their use from spreading to a larger extent in microgrids. These challenges include low energy density, high economic costs, and thermal management [110]. While supercapacitors offer high power density, their low energy density limits their deployment in larger-scale applications. The energy densities of supercapacitors are not very high. There is a gap in the energy density between supercapacitors (<20 Wh kg^−^^1^) and batteries (30–200 Wh kg^−^^1^), and research and study are still underway to improve the efficiency of supercapacitors. To effectively improve the capacity of supercapacitors, the manufacturing process and material technology have been improved, but in the long run, it has been difficult to find new active materials for electrolyte and electrode with better electrochemical performance. In order to improve the energy densities of supercapacitors, it is necessary to increase the effective surface area of the electrode materials in double-layer capacitors. If these areas are properly treated, the energy densities of supercapacitors can become comparable to batteries. The economic cost of supercapacitors remains relatively high compared to some other energy storage technologies. In addition, effective thermal management remains critical to ensuring the efficient performance and long-term safety of supercapacitors.

To address these challenges, researchers and various studies are focusing on developing electrodes using new materials with higher energy density. They are also maximizing the efficiency of energy conversion processes, overcoming thermal management issues and improving new technologies for this purpose, and cost-effective manufacturing of supercapacitors. By overcoming these challenges, supercapacitors and HESS have the potential to revolutionize the energy storage landscape and play an important role in the transition to a sustainable energy future.

#### 5.4.2 For HESSs topologies

In addition to the many advantages of hybrid ESS configuration and their integration into MGs, there have been recent developments in this field that support the idea of applying hybrid configurations to electrical grids, including power management algorithms and modular and scalable designs [111]. Algorithms of advanced control such as MPC and adaptive control strategies are being implemented to manage the interactions between different storage types effectively. These algorithms help distribute energy according to the operational state of the system, optimizing performance and lifespan. Research into modular HESS architectures has made it easier to scale up or down depending on the application. Such designs can integrate with distributed energy resources, like solar panels and wind turbines, and provide efficient energy storage for microgrids.

However, despite the advantages and developments that may make the hybrid configuration one of the best configurations, as it combines the advantages of different storage systems such as batteries, SCs and fuel cells with renewable energy sources such as solar cells and wind energy, there are some challenges that make it difficult to implement such systems in practice. These challenges can be summarized as complex control strategies, compatibility and integration, cost and space. Integration of multiple storage types requires complex EMS for real-time control, which can increase the system's complexity and development costs. Moreover, ensuring that different storage technologies can work together without issues, such as differences in voltage levels or power characteristics, remains a significant challenge. The need for both battery and supercapacitor modules, along with power converters and control systems, can increase the cost and spatial footprint of HESS.

## 6 Energy management systems (EMSs) for ESS

In HESS, the most crucial problem is creating and implementing a suitable control system. This depends on various parameters as follows [85]:

• The purpose of using HESS for example improving intermittency, power quality, or storage lifetime.
• System type (AC-MG, DC- MG, grid-connected).
• Control method cost.
• Response time of control method.
• Hybridization architecture.

In HESSs, it is necessary for operation a stable, safe, and efficient system. Yet, a feasible power-sharing plan is more significant. In general, it can be classified the management and control of HESS as follows:

• The subordinate control unit that regulates the current or power flowing through the HESS components uses a reference signal produced by the subordinate control unit. This is for energy management.
• The energy management component that distributes power across the HESS storage units to improve system dynamics, achieve high overall efficiency, monitor SOC, lower system loss and costs, and decrease operational

The control goals of (HESS) can be classified in standalone MG into three categories [72]:

• Optimization of MG performance.
• Increase reliability and stability for system.
• Reduce the cost of set-up and operating.

**Fig. 25:**
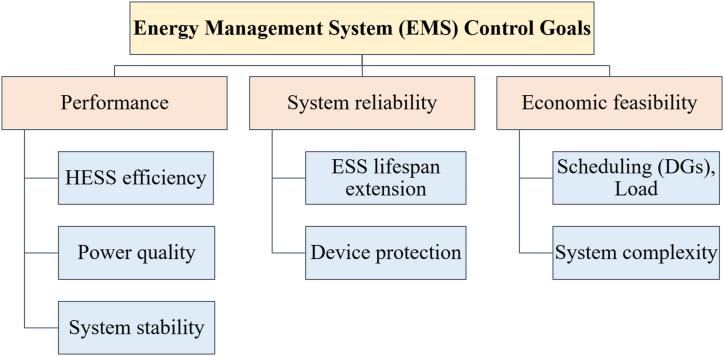
Summarizes goals of EMS control.

**Fig. 26:**
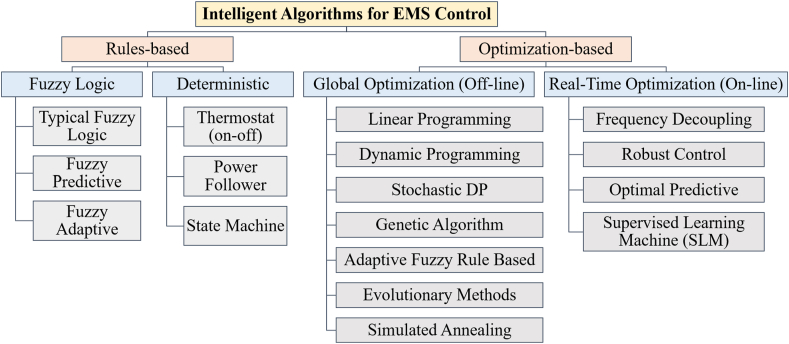
Intelligent algorithms categorization for EMS supervisory control in (HESSs) summarized.

Fig. 25 classifies EMS control goals. An active HESS topology enables each ESS unit to be optimized through an EMS. The purpose of the EMSs is to maximize the advantages of HESSs. It is necessary to maximize volumetric and columbic efficiency while preserving system reliability. In order to maintain system dependability, EMSs should provide reliable system performance under all scenarios of loading, safeguard the ESSs from harsh environments, and increase the usable lifetime of the ESSs components. The implementation, operating, and maintenance costs of EMS must be maintained to a minimum as illustrated in Fig. 26. Classify intelligent algorithms for EMS supervisory control in a succinct manner [72].

### 6.1 EMSs structure

**Fig. 27:**
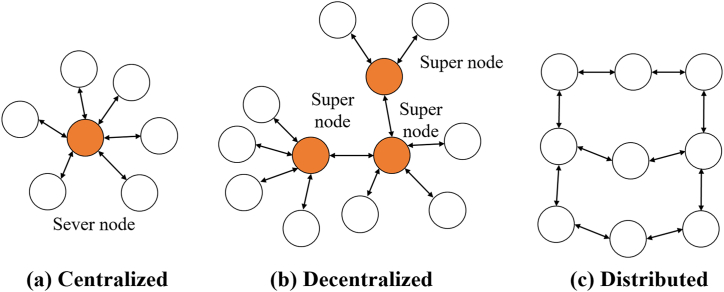
EMSC categories.

EMSs perform the functions necessary for the efficient operation of electricity generation and transmission operations to ensure safe energy supply while ensuring economical and low costs. The EMS performs a set of operations that include data analysis, predictions, optimizations, and a human-machine interface (HMI). EMS in MGs is a complex system it works automatically. That aims to coordinate and optimally schedule available resources (main grid, DG, and ESS) to meet load requirements taking into account changes in weather data and sudden changes in loads. As shown in Fig. 27, EMSC in microgrids are classified to three categories (a) centralized, (b) decentralized, and (c) distributed as depicted in Fig. 27(a) and (b) and (c) respectively. Which shows its characteristics in Table 12 [112].

**Table 12:** Characteristics of EMSC categories.

Characteristics	Centralized	Decentralized	Distributed
Information Accessed	Microgrids pass information to the central controller	Independent control is provided with data from the other local controllers	Interoperability and data exchange between every device
Communication Information	Synchronized information from the device to the central controller	Information among local controllers is asynchronized	Communication is both locally and globally asynchronized
Function in real-time	Complex	Acceptable	Easy
Feature of Plug and play	The central controller needs to be instructed	Can be accessed by central controller	Available by the peers
Expenditure	More	Less	Less
Structure of Grid	Centrally controlled	Locally controlled	Both centrally and locally controlled
Tolerance during fault	Less tolerance capability	One router fault—tolerated	N router fault—tolerated, Possible self-healing feature
Operation Flexibility	Very less	Available	Very much needed
Safety measures	Less	Available	High

### 6.2 EMSs and SCs integration in HESSs

The advantage of integrating SCs with HESSs and controlling them with advanced energy management strategies enhances ESS flexibility, efficiency and stability. Recent developments in materials science, energy management algorithms and the integration of AI with management systems have led to the development and improvement of the response between these systems and different applications [113]. However, there were some challenges that required future research studies to be able to be deployed on a larger scale in microgrids. EMSs are a critical component of HESS operation, coordinating the flow of energy between batteries and SCs to achieve maximum efficiency and sustainable use of resources. The main functions of EMS in HESS can be summarized as charge and discharge management, and advanced control strategies, and adaption to grid changes. In fact, EMS monitors the SOC of SCs and batteries, and decides when to draw power from each device or charge it. In addition, advanced control strategies such as model predictive control (MPC) and rule-based control, ensure optimal use of stored power based on expected loads and energy availability. EMS can respond dynamically to changes in demand or production from renewable sources, such as solar and wind. This increases the system's ability to cope with grid fluctuations.

### 6.3 Recent advancements in EMS for HESS

With the increasing development in the use of ESSs and the continuous need for strategies to organize and manage these systems, this in conjunction with technological development and the spread of AI in applications, there have been developments that have appeared in MG management systems and storage systems with their various configurations [114]. The most prominent of these developments are AI integration in control systems, distributed control systems, and protocols for advanced communication [113]. The integration of machine learning (ML) algorithms in EMS allows systems to predict changes in load demand and generation patterns and helps in optimal utilization of storage. These algorithms support real-time power flow adjustment and adaptation to fluctuations under different operating conditions. Moreover, advanced EMS can operate in a distributed manner, where local controllers can manage energy storage at different points distributed in the grid, enhancing the flexibility and responsiveness of the systems. The use of IOT devices and communication standards such as Modbus and IEC 61850, allows for seamless data exchange and coordination between different components of the energy storage system.

### 6.4 Challenges in integrating of EMSs

There are some challenges facing the integration of EMSs, with all their complexities and configurations. These challenges require future research studies to be able to be deployed on a wider scale in microgrids. These can be summarized as real-time data processing, cybersecurity and cost and development time [115]. Processing large amounts of data in real-time to make immediate decisions in emergency situations and rapid changes in the power system requires a large amount of computing power and requires high-performance hardware and software solutions. With the increase in communications in modern EMSs, it has become important to secure the system against any cyber threats, which is of paramount importance to prevent any data breaches or manipulation of control. Implementing advanced EMSs that can be seamlessly integrated with emergency management systems and support adaptive algorithms requires a significant investment in research and development and can extend the project development timeline.

## 7 Micro-grid different operating modes

Black-start mode, island mode, grid-connect start mode, and grid-connect mode are the major four operating modes for the MG [[55], [98], [116]]. Accordingly, each subsystem operates in the appropriate manner during various MG states, as shown in Table 13.

**Table 13:** Operation stats under different operating condition.

Operation state of micro-grid state	Operation mode of each subsystem		
	PV	Li-PCS	SC-PCS
Black-start	Power off	VF	PQ
Island state	MPPT	PQ	VF
Grid-connect start state	Power off	PQ	PQ
Grid-connect state	MPPT	PQ	PQ

### 7.1 Start mode

An unexpected black-start procedure is anticipated in MG island mode. The SOC of a Li-PCS must be over 80% for this procedure to begin; otherwise, the MG will switch to the grid link start step. A SC-PCS runs in mode of PQ for pre-charge while Li-PCS activates in mode of VF to supply the MG voltage during the black-start procedure. The TSS will turn on if Li-PCS is insufficiently charged to start the MG, charging Li-PCS using the power grid. Before the MG stops for the last time, the energy management method typically keeps SOCLi over 80%.

### 7.2 Island mode

As previously indicated, the MG is request to utilize the internal energy before switching to grid-connect mode. Due to its quick responsiveness to changes in power, SC-PCS functions as a VSI in these circumstances. Li-PCS runs in PQ mode to control SC-PCS's power flow. Under MPPT management, PV production systems will consistently provide the greatest power. Interruptible loads will be isolated if the power supply and energy can't keep up with the demand from the load. After isolating all interruptible loads, if there is still insufficient energy and power, the MG will switch to on-grid mode.

When a fault or other type of power disruption occurs when operating in an island-based mode, the MG disconnects from the main grid. Creating a MG by separating a portion of the electrical grid, which consists of DG units and loads, from the rest of the grid. This makes it necessary for the islanded MG to offer auxiliary services in order to function in a steady and regulated way. The DG equipment must recognize islanding when the main grid is cut off from the MG. In this case, one of the controllers has to be set to control mode of VF. The network drops its Slack reference during islanding, which is later recognized by a VF controller.

When the transmission grid is cut off, the frequency and voltage for the islanded MG vary, and these variations are brought on by the load-generation imbalance. Here is where supercapacitors play a crucial role. If the exceeded/desired quantity of active and reactive power is absorbed/injected by the locally accessible sources in the islanded MG, the voltage and frequency can be returned to the allowable limits [117]. However, managing the energy flow between renewable energy sources and ESSs while they operate in various modes is a difficult challenge [87].

### 7.3 Grid connected mode

Grid-connected state is never utilized to transport power from the MG to the electrical grid; rather, it is solely used to charge HESS. Both SC-PCS and Li-PCS are in PQ mode in this situation. When SOCLi has recovered to 95%, the MG will revert to an island state. Grid-connected mode is the MGs typical operating mode. All of the feeders are being supplied by the utility or the main grid in this operating mode. Without affecting the main grid's power quality, the MG runs smoothly.

In contrast to the grid-connected mode, in the islanded mode, the DG unit control system development is different. The on-grid mode control system may be set up in either P-V or PQ modes, depending on the situation, and the dependents on it will be handled accordingly. If the control system's objective is to control the active power and voltage of the units of DG, it is necessary to create in P-V mode. If the goal is to maximize the supply of active power by managing the units' reactive power, the system of control must be designed in mode of PQ [117].

## 8 Artificial intelligence (AI) in microgrids

AI is a vital tool in the efficient management and operation of microgrids, relying on big data analysis and pattern extraction to guide optimal operations. Through machine learning and deep learning algorithms, AI can improve several aspects of microgrids, such as [[100], [101], [118], [119]]:

• Smart energy management: AI dynamically predicts and manages energy needs, optimizing energy distribution between renewable energy sources (such as solar and wind) and other systems such as batteries and supercapacitors.
• Demand forecasting: AI can predict energy consumption based on historical data and consumption patterns, helping microgrids avoid overloads and reduce losses.
• Fault detection and self-repair: AI can detect potential problems in a microgrid early, such as faults in electrical systems or components, and deal with them before major failures occur.

### 8.1 Integration of AI and supercapacitors in microgrids

Integration of AI and supercapacitors in microgrids brings several benefits that enhance system efficiency and reliability [[120], [121]].

• Improved storage efficiency: Through AI algorithms, the utilization of supercapacitors can be optimized by predicting optimal operating conditions. AI can schedule charging and discharging operations based on energy consumption patterns, reducing waste.
• Power flow management: AI can optimize how the energy stored in supercapacitors is distributed among different devices in the microgrid, ensuring that energy is used efficiently and according to priorities.
• Capacitor performance prediction: AI can predict the performance of supercapacitors by monitoring energy consumption and charging and discharging rates, thus predicting when to replace capacitors before the overall performance of the grid is affected.

### 8.2 Efficient energy management using AI and supercapacitors

**Fig. 28:**
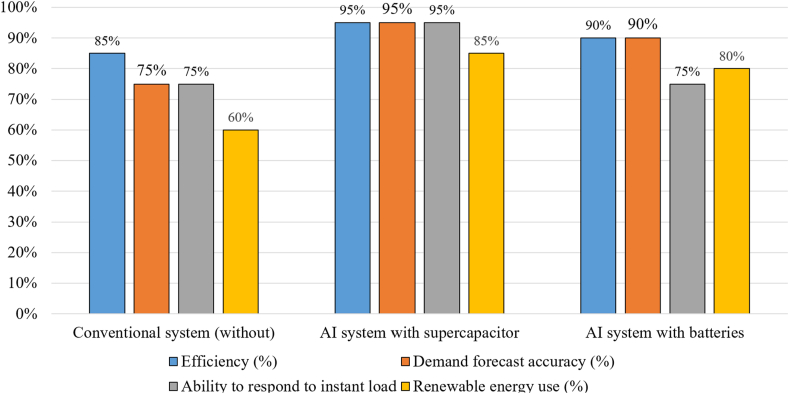
Efficiency determination criteria for different types of MGs.

**Fig. 29:**
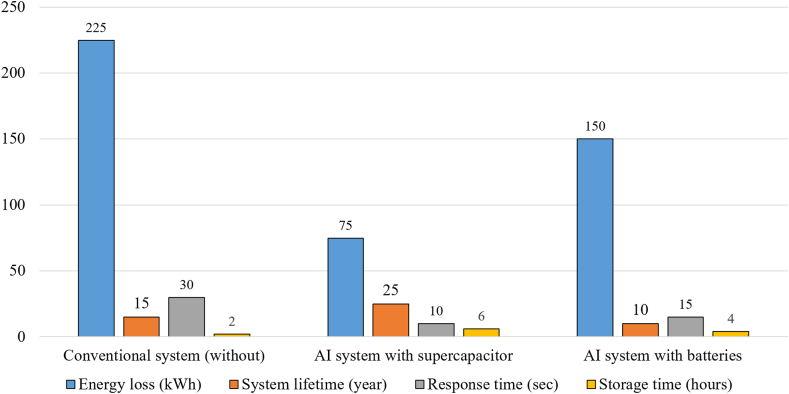
Losses (KWh), life time, response time and storage time of different types of MGs.

**Fig. 30:**
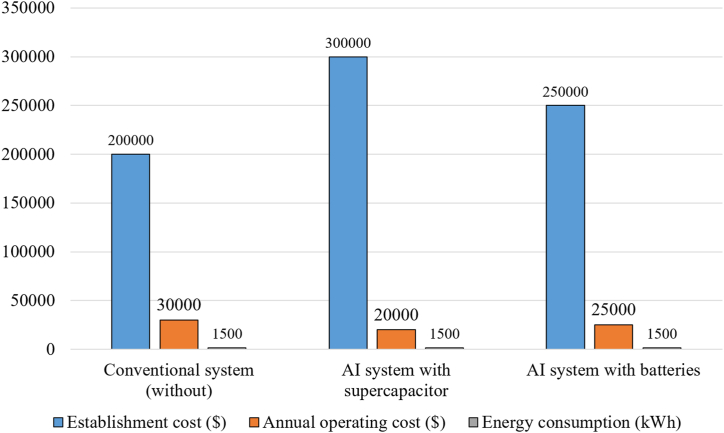
Costs and energy consumption of different types of MGs.

The integration of AI and supercapacitors in microgrids leads to improved efficiency and flexibility, with increased reliance on renewable energy and reduced losses. This is determined by a set of factors such as the speed of response to loads, foundational and operational costs, expected life and storage time. This is illustrated in the comparisons shown in Fig. 28, Fig. 29, Fig. 30 [[122], [123]].

## 9 Analysis and discussion

### 9.1 Authors' suggestions

**Fig. 31:**
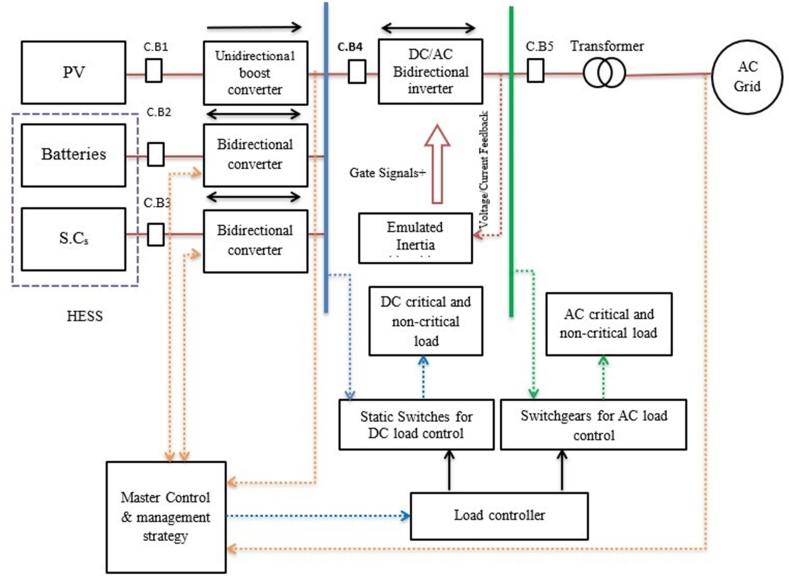
A proposed system for an HMG based (HESSs).

In this part of the paper, the authors propose a system for a MG that relies on a renewable energy source as shown in Fig. 31, which is solar energy cells (PV) to feed the loads consisting of DC loads and AC loads. Others that support the system, such as converters (boost, buck-boost), bi-directional inverters, control circuits, and energy management. It is also proposed to connect with the grid. This proposed system mainly aims to stabilize the operating system in various conditions, such as black start mode, optimize system inertia, meeting demand and variation of loads, and transient variation of frequency. Here comes the importance of HES. This design will be studied and researched in another research paper by the same authors and its results will be presented later.

### 9.2 Summary of review authors' opinions

Through what was presented of literature and previous studies during this review, the review authors adopted the idea of supercapacitors for use it is in the MG as following.

• As an actionable solution to fine MG issues.
• As a flexible and scalable support for the MG in many operational conditions and requirements.
• These opinions also agree with the opinions of other researchers are based on the advantages offered by supercapacitors, which could be improved in the future such as This was the answer to a very important question: why supercapacitors?
• The microporous structure of the electrodes allows for a higher storage capacity compared to conventional capacitors or batteries.
• Rapid response when entering the grid in various emergency operational conditions such as black start, improving grid inertia and overloading demand.
• Fast charging and discharging time, which allows for a large number of (charging – discharging) cycles in a short time while maintaining its high performance and efficiency, as well as its lifetime, unlike batteries.

## 10 Limitations and future trends

From all of the above, this paper recommends the development of supercapacitors until reaching high capacities. This may be a feasible future task, especially with the emergence of nanotechnology. Also recommend researchers work on achieving ESSs that achieve total dependence on renewable sources in electrical networks. This will completely dispense with systems conventional generation based on synchronous generators.

## 11 Conclusion

Based on what was reviewed in previous studies and literature, it became clear that there are issues and challenges around the electric power grid, and the matter becomes more difficult when MGs exist in different operating conditions (connected mode or islanded mode), especially when relying on RES such as solar or wind energy, which suffer inconvenience due to its dependence on fluctuating weather conditions. This is in addition to operating problems such as faults, overloading, economic problems, failure to meet power demand, and others that are considered burdens that prevent the grid stability and reliability. A solution to problems and challenges was the emergence of the ESS. Therefore, this review focused the light on supercapacitors as a promising and future solution with explained (Working Principles and Specifications, Modeling, Classification, Materials Lifetime expectancy, Integration with MG as storage system into (HESSs) and (HESSs) topologies) and it was necessary, in order to complete the topic, that (MG with (HESS) based on (AC or DC) and (hybrid AC–DC) bus, (EMSs) for ESSs and MG Different Operating Modes) be clarified. In light of this, it was concluded that ESSs based on super capacitors, especially hybrid systems used with MGs, have benefits from them as follows:

• Optimize storage and system lifetime.
• Improve overall system efficiency.
• Reduction of total investment costs.

The integration of AI, SCs and MGs represents a paradigm shift in the energy sector. This integration provides advanced solutions for more efficient energy management and better storage, which enhances the sustainability and stability of microgrids.

## CRediT authorship contribution statement

**Mohamed El-Sayed M. Essa:** Writing – review & editing, Writing – original draft, Visualization, Validation, Methodology, Investigation, Formal analysis, Data curation, Conceptualization. **Mohammed Fouad Ali:** Writing – review & editing, Writing – original draft, Visualization, Validation, Software, Methodology, Investigation, Formal analysis, Data curation, Conceptualization. **Elwy E. El-kholy:** Writing – review & editing, Writing – original draft, Visualization, Validation, Resources, Methodology, Investigation, Formal analysis, Data curation, Conceptualization. **Mohammed Amer:** Writing – review & editing, Writing – original draft, Visualization, Validation, Supervision, Software, Project administration, Methodology, Investigation, Formal analysis, Data curation, Conceptualization. **Mahmoud Elsisi:** Writing – review & editing, Writing – original draft, Visualization, Validation, Methodology, Investigation, Formal analysis, Data curation, Conceptualization. **Uzair Sajjad:** Writing – review & editing, Writing – original draft, Visualization, Validation, Methodology, Investigation, Formal analysis, Data curation, Conceptualization. **Khalid Hamid:** Writing – review & editing, Writing – original draft, Visualization, Validation, Methodology, Investigation, Formal analysis, Data curation, Conceptualization. **Hilmy El-sayed Awad:** Writing – review & editing, Writing – original draft, Visualization, Validation, Methodology, Investigation, Formal analysis, Data curation, Conceptualization.

## Data availability

Data will be made available on request. For requesting data, please write to the corresponding author.

## Declaration of competing interest

The authors declare that they have no known competing financial interests or personal relationships that could have appeared to influence the work reported in this paper.

## Figures and Tables

**Table:** 

Abbreviations	Detailed terms	Abbreviations	Detailed terms
IRENA	International Renewable Energy Agency	EMS	Energy management system
PV	Photovoltaic	HES	Hybrid energy storage
SC	Supercapacitor	HESS	Hybrid energy storage system
ESS	Energy storage system	HSC	Hybrid supercapacitor
ESSs	Energy storage systems	BSHS	Battery Supercapacitor Hybrid Storage
A	Area of electrode surface	MG	Microgrid
$\varepsilon_{0}$, $\varepsilon_{r}$	Free space permittivity, Relative permittivity of the dielectric material	HMG	Hybrid microgrid
d	The distance between two oppositely biased electrodes	PCS	Power conversion system
C	The capacitance of the capacitor	SCSS	Supercapacitor storage system
ESR	Equivalent series resistance	BESS	Battery energy storage system
SI	Series inductance	FC	Fuel cell
EPR	Equivalent parallel resistor	VS	Voltage source
EDLC	Electrolytic double layer capacitor	CS	Current source
VSI	Voltage source inverter	SOC	State of charge
VF	Voltage-frequency	TSS	Thyristor static switch
PQ	Active/reactive power mode	SC-PCS	Supercapacitor power converter system
DG	Distributed generation	Li-PCS	Lithium-ion battery power converter system
P-V	Power voltage mode	CAES	Compressed Air Energy Storage
RES	Renewable Energy System	AI	Artificial Intelligent
HMI	Human-machine interface	EMSC	Energy management system control
IOT	Internet of Things		
